# Randomised controlled trial to measure effectiveness and cost-effectiveness of a digital social intervention promoted by primary care clinicians to adults with asthma to improve asthma control: protocol

**DOI:** 10.1136/bmjopen-2025-104367

**Published:** 2025-09-12

**Authors:** Georgios Dimitrios Karampatakis, Helen E Wood, Chris J Griffiths, Stephanie JC Taylor, Veronica Toffolutti, Victoria J Bird, Nathan C Lea, Richard Ashcroft, Neil S Coulson, Pietro Panzarasa, Xiancheng Li, Aziz Sheikh, Clare Relton, Nishanth Sastry, Jane S Watson, Jonathan Mant, Viv Marsh, Bill Day, Borislava Mihaylova, Neil Walker, Anna De Simoni

**Affiliations:** 1 Centre for Primary Care, Wolfson Institute of Population Health, Barts and The London School of Medicine and Dentistry, Queen Mary University of London, London, UK; 2 Centre for Applied Respiratory Research, Innovation and Impact, Queen Mary University of London, London, UK; 3 Department of Medical Informatics & Statistics, The European Institute for Innovation through Health Data, Ghent University Hospital, Ghent, Belgium; 4 The City Law School, City St George’s, University of London, London, UK; 5 School of Medicine, University of Nottingham, Nottingham, UK; 6 School of Business and Management, Queen Mary University of London, London, UK; 7 Nuffield Department of Primary Care Health Sciences, University of Oxford, Oxford, UK; 8 Surrey Centre for Cyber Security, Department of Computer Science, University of Surrey, Guildford, UK; 9 Respiratory Department, St George’s Healthcare NHS Trust, London, UK; 10 Department of Public Health and Primary Care, University of Cambridge, Cambridge, UK; 11 Usher Institute, College of Medicine and Veterinary Medicine, The University of Edinburgh, Edinburgh, UK

**Keywords:** primary care, asthma, self care, social media, protocols & guidelines, randomized controlled trial

## Abstract

**ABSTRACT:**

**Introduction:**

In the UK, approximately 5.4 million adults live with asthma, of whom one in five have an uncontrolled form. Uncontrolled asthma reduces quality of life and increases healthcare use. Engaging with peers through online health communities (OHCs) can empower patients to self-manage their long-term condition. While OHCs have been in existence for several years and growing numbers of patients access them, the role of primary care in signposting patients to them has been minimal and ad hoc. We have co-developed with patients and healthcare professionals (HCPs) an intervention for adult patients with asthma, consisting of an appointment with a primary care HCP to introduce online peer support and sign patients up to an established asthma OHC, followed by OHC engagement. Feasibility work found the intervention acceptable to patients and HCPs. This protocol outlines our plan to test the intervention’s effectiveness and cost-effectiveness.

**Methods and analysis:**

An individual randomised controlled trial will be carried out. Eligible participants will be recruited via an online survey sent to adult patients on the asthma register in 50–70 general practices in several UK locations. Participants will be invited to attend a one-off, face-to-face appointment with a primary care HCP, during which they will be individually randomised to the intervention or usual care. An asthma control test (primary outcome) and other measures of clinical effectiveness will be collected at baseline and every 3 months over a 12-month follow-up period. Descriptive and inferential statistics will be used to compare outcome measures between study arms. Cost-effectiveness assessment of the intervention compared with current standard of asthma management in primary care will be reported. A sample of patients and HCPs will be interviewed at study exit and the data analysed thematically.

**Ethics and dissemination:**

The study was approved by a National Health Service Research Ethics Committee (reference: 25/NE/0006). Written consent will be obtained from all participants. Findings will be disseminated through various means, including sharing with general practices, conference presentations and peer-reviewed publications.

**Trial registration number:**

NCT06849245.

**STRENGTHS AND LIMITATIONS OF THIS STUDY**

- The study will employ a robust mixed-methods design within a definitive randomised controlled trial (RCT) to examine the relationship between a digital social intervention promoted by primary care clinicians and outcomes.
- By applying qualitative, social network and sentiment analysis methods on data provided by the hosting platform of an online health community (OHC), it will be possible to thoroughly study patients’ engagement with the OHC.
- The study will recruit participants who are proficient in English and digitally skilled; therefore, the sample may not be fully representative of the wider asthma patient population.
- The primary outcome of interest, asthma control test (ACT), may be influenced by co-existing non-asthma-related conditions and can remain low despite good asthma control; therefore, we will triangulate ACT scores with other outcomes, especially asthma-related healthcare utilisation.

## Introduction

 In the UK, approximately 5.4 million adults live with asthma.^1^ About one in five of the adult population with asthma experiences an uncontrolled form of asthma.^2^ Uncontrolled asthma results in 6.3 million primary care consultations^3^ as well as 60 000 emergency hospital admissions and 200 000 hospital bed days per year.^4^ Asthma-related care costs the National Health Service (NHS) approximately £1.1 billion per year.^3^ Within the UK, mortality due to lung-related conditions is the second worst in Europe after Turkey,^5^ with 12 000 people dying from asthma between 2014 and 2024.^6^

Uncontrolled asthma often leads to comorbidities, including symptoms of anxiety and/or panic and loss of social connections,^7^,^9^ as well as lower quality of life and reduced ability to perform daily activities.^10^ Patients with uncontrolled asthma often enter a vicious cycle in which associated psychological effects and loneliness negatively influence perception of symptoms and ability to self-manage their condition, thereby further worsening self-efficacy and control of asthma.^8^,^10^

Patients’ asthma control, and associated morbidity and mortality, could be enhanced by supporting and improving self-management skills.^11^,^13^ It has been reported that engaging with peers (ie, others with similar health-related problems and/or concerns) through online health communities (OHCs) improves patients’ self-efficacy and illness understanding, thus empowering them to take responsibility for their own health and fostering their ability to cope.^14^,^17^ OHCs are increasingly being used as a means of offering and receiving online peer support.^18^ Considering their widespread adoption, OHCs could promote self-management of patients with long-term conditions at scale, thereby addressing NHS initiatives to support self-management in large cohorts of patients.^19^

There is a need for additional research on peer support interventions to establish effectiveness, acceptability and effects on health-related outcomes and inequalities.^20 21^ To date, a few studies have reported that anonymous interactions with a range of people and intuitive access to resources and tailored information and emotional support are the main benefits of engaging with an OHC.^1622^,^28^ The vast majority of claims about the usefulness of OHCs, however, are based on qualitative studies,^24 29^ while quantitative assessments of OHCs have mainly focused on studying intervention mechanisms without necessarily measuring clinical outcomes from OHC engagement.^30^,^35^

Using OHCs to access lay advice from peers is not a new concept. However, signposting patients to OHCs and online peer support as part of primary care services is a novel strategy.^36^ Previous research found that when healthcare professionals (HCPs) refer patients to studies testing digital interventions, retention rates significantly increase.^37^ It is also known that, as the primary care setting is usually the first point of contact for healthcare purposes, long-term relationships and trust are often formed between primary care clinicians and patients.^38^

We therefore envisaged the idea of primary care HCPs enabling patients who might benefit from online peer support. Together with primary care HCPs and patients with asthma, we developed the content and delivery of a two-part primary care digital social intervention.^39^ The first component consists of a structured, face-to-face appointment for adults with uncontrolled asthma, during which primary care HCPs introduce patients to online peer support and sign them up to the asthma OHC of the Asthma+Lung UK (ALUK) charity. The second component consists of actual engagement with the OHC. The ALUK asthma OHC is a well-established asynchronous text-based health forum that has around 20 000 registered and 2000 active users (ie, people who regularly use the OHC). The forum is hosted by HealthUnlocked and is moderated by HealthUnlocked staff and specialist respiratory nurses employed by ALUK.

We conducted a study to test the feasibility and acceptability of the recruitment strategy and intervention.^40^ This work demonstrated that intervention delivery was feasible and acceptable to patients in primary care settings. Patients showed, as expected, varied levels of OHC engagement. The feasibility study enabled us to refine the intervention by considering various practicalities around recruitment and delivery (eg, the importance of delivering the intervention as soon as possible following recruitment for patients to maintain enthusiasm and interest). The current protocol outlines a plan for a definitive trial to test the effectiveness of our intervention and assess its cost-effectiveness. The results will provide robust evidence regarding the benefits of promoting online peer support as part of primary care services for patients with asthma. If the intervention is proven effective and scaled up, it will enable primary care HCPs to offer it as part of routinely offered asthma services in primary care.

### Methods and analysis

### Study design

**Figure 1:**
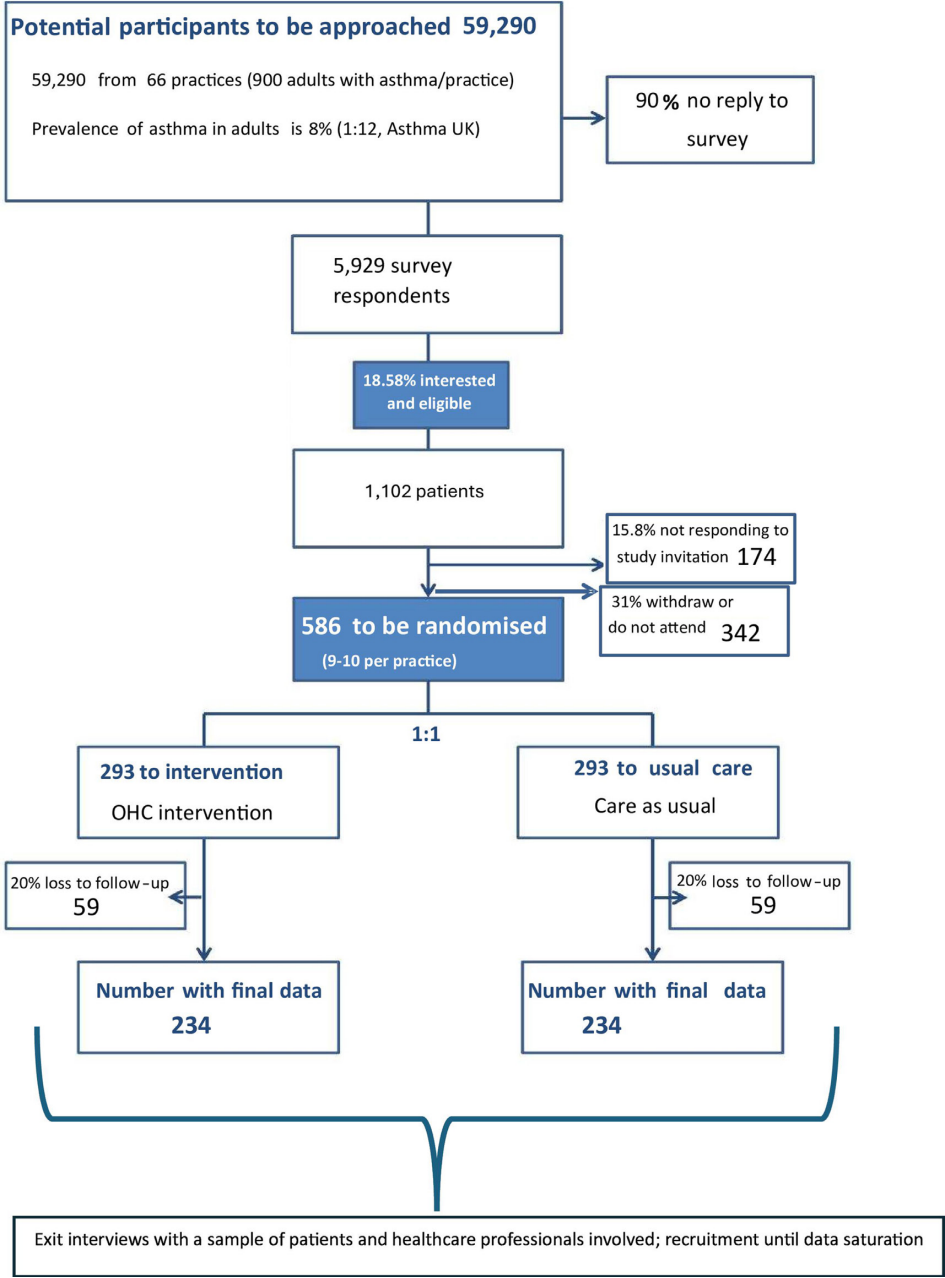
Flow diagram summarising the randomised controlled trial process. OHC, online health community.

‘A survey leading to a trial’ (ie, recruiting survey to identify and invite to the trial people interested in online peer support) was selected as the best design to assess an intervention that is only likely to appeal to a subset of the asthma patient population (ie, those interested in online peer support). A randomised controlled trial (RCT) will be conducted. There will be four steps in the study: (i) a survey to identify and recruit eligible patients; (ii) a structured, face-to-face appointment to deliver the intervention or provide usual care; (iii) collection of follow-up outcome measures and (iv) one-to-one exit interviews with a sample of patients and primary care HCPs. Figure 1 schematically summarises study procedures.

### Participants

Approximately 600 patients with uncontrolled asthma (ie, an asthma control test (ACT) score of <20) from 50 to 70 general practices will be recruited via an online survey, sent to approximately 60 000 patients on the asthma register.

To participate in the study, participants will need to:

- Be adults (aged 18 years and above) on the asthma register, who have expressed their interest in digital social interventions in the recruitment survey;
- Report uncontrolled asthma in the recruitment survey;
- Be able to provide informed consent, as determined by the HCP carrying out the study appointment.

Exclusion criteria

Patients who are:

- Already members of the ALUK asthma OHC or other asthma OHCs/Facebook groups (general use of social media will not prevent participation).
- Receiving palliative care or with very limited life expectancy (<12 months).
- Receiving institutional long-term care (receiving total care in residential homes or living in nursing homes).
- Considered unsuitable to take part in the study by their general practitioner (GP)/nurses.

### Recruitment processes

Assistance in identifying general practices willing to act as recruitment sites will be sought from four National Institute for Health and Care Research Regional Research Delivery Networks (NIHR RDNs): North London, South London, South Central and East of England); further RDNs may be approached, if necessary. Nurses’ groups (eg, from the Primary Care Respiratory Society, or London Respiratory Nurses) might also be approached to identify practices interested in joining the study.

All eligible adults on the asthma register will be identified through searches on the clinical record systems (performed by practice-based staff) at the general practices. A text message will be sent to patients directly from the clinical record system or through AccuRx (ie, an NHS-approved software provider used by primary care professionals to contact their patients). This text message will include a link to a short online survey. The survey will act as a recruitment tool and will also include a link to the Participant Information Sheet (PIS). Patients will be given a deadline of 1 month to fill in the survey and a single reminder text message will be sent, 1 week before the deadline. Posters and/or leaflets will also be displayed at participating general practices, including a QR code link to the recruitment survey.

Eligible patients will be invited to a structured, face-to-face appointment with a primary care HCP to receive the intervention or usual care. Invitation and relevant arrangements for the appointment will be made by general practice staff supported by the research team (through text messages/phone calls/emails). A mutually convenient time for the HCP and the patient will be established.

### Study procedures

#### Recruiting survey

The survey includes a screening section, asking patients to confirm they have an asthma diagnosis and are over 18 years, followed by questions establishing their eligibility for the trial (ACT questionnaire, membership of online asthma forums/Facebook groups and interest in taking part in the trial), with an additional section about support with managing their asthma. Eligible respondents will be asked to provide their contact details. Survey and study data will be recorded using Research Electronic Data Capture (REDCap) software, a secure application for designing and managing online surveys and databases. Completion of the recruiting survey is anticipated to take <5 min (see online supplemental material 1 for a copy of the recruiting survey).

#### Structured appointment

All participants will be invited to attend a one-off, face-to-face appointment with a primary care HCP (most likely a general practice-based nurse or pharmacist) at their general practice, lasting 30–45 min depending on whether they receive the intervention or usual care only, where baseline data collection and randomisation will be undertaken. Participants will be individually randomised to either receive the intervention or to receive usual care. Those allocated to the intervention arm will be introduced to the intervention (see 'Intervention arm' section below). All HCPs involved will complete an online training course designed by the research team, covering all aspects of the face-to-face appointment delivery (including data capture in REDCap, randomisation, how to explain the control arm of the trial and how to introduce online peer support and motivate intervention arm participants to engage with the OHC).

#### Randomisation

The randomisation ratio to both treatment arms will be 1:1. During the face-to-face appointment, the HCP will use a randomisation tool embedded in the study database on REDCap to determine group assignment (intervention or control) for each participant. Randomisation lists will be prepared prestudy commencement using a permuted block approach in which the number of allocations is 50:50 within each block, the size of which is determined randomly from possible block sizes of 4, 6 or 8. The sequence of allocations within a block is randomly permuted, conditional on the 50:50 allocation requirement (so a block of size 4 gives rise to the following six possible sequences of allocations: AABB, ABAB, ABBA, BAAB, BABA and BBAA). Allocation will be stratified by asthma severity, based on ACT score (two groups: lower (ACT <12), higher (ACT ≥12) to 19) and age (two groups: low (age 18–49 years), high (age 50–99 years)) such that each stratum (ie, combination of asthma severity group and age group) has its own randomisation list, so ensuring balance of these factors between the treatment groups. Blinding will not be possible for HCPs and patients. The central research team will also be unblinded as members will need to contact patients for interim follow-ups and reminders.

#### Intervention arm

After the collection of baseline measures, the face-to-face appointment with participants in the intervention arm will involve:

- Signposting to the ALUK asthma OHC by offering a ‘tour’ of the OHC and explaining the different sections of the website.
- An introduction to norms and values for passive (just reading) and active (writing OHC posts) participation.
- Explaining the differences between posting publicly and privately (public posts are shared, according to HealthUnlocked terms and conditions, with third parties whereas private messages can be sent via the chat function and are not shared).
- Motivation for engagement with the OHC to seek and offer self-management information and support, by emphasising that the OHC could be used ad hoc (eg, when feeling unwell or when emotional support is needed).
- Problem solving with respect to any difficulties/concerns.
- Signing patients up to the OHC, by explaining terms and conditions of HealthUnlocked.

To sign up participants, HCPs will ask them to use their own mobile phone/other electronic device and access a study-specific landing page provided by HealthUnlocked, outlining the consenting process to the OHC and containing a privacy notice. This allows easy onboarding by default opting out participants from any sharing of collected data with third parties. Participants will need to provide their email address and will be invited to choose an anonymous username. Participants’ email addresses will not be visible within the OHC.

Participants will leave the face-to-face appointment with a card with a QR code link to the ALUK OHC on one side and to the study website/contact details on the other side and a reminder of their username and password (data not recorded for the study). Once logged into the OHC, participants will have the opportunity to change their username/password as well as any other settings in their account (eg, opting in for receiving HealthUnlocked emails).

Depending on the availability of resources, monthly text messages might be sent to participants who have consented, reminding them to engage with the OHC.

#### Control arm

After the collection of baseline measures (same measures to the intervention arm), control group patients will go back to receive usual care only without any reference to OHCs and online peer support.

### Outcomes and measures

Twelve months following the face-to-face appointment, all participating patients will receive a text message and/or email with a link to an online survey collecting follow-up measures (up to three reminders will be sent). In addition, patients who have consented to receiving regular contact will be telephoned by a member of the research team every 3 months during the 12-month follow-up period to collect data on asthma control, quality of life and care utilisation.

The primary outcome of interest will be ACT score collected every 3 months for 12 months following the face-to-face appointment (adjusted for baseline value in the analysis). Secondary outcomes will include variables related to the overall health and well-being of the patient, as well as associated care utilisation (table 1). In addition to outcomes self-reported by patients (table 1), non-self-reported outcome variables will be obtained (table 2), depending on data availability and consent for access to healthcare records.

**Table 1:** Outcomes self-reported by participants

Domain	Outcome	Measure	Baseline*	3 months†	6 months†	9 months†	12 months‡
Clinical factors	Control of asthma§	ACT questionnaire	X	X	X	X	X
	Asthma exacerbations over last 3 months	Bespoke question	X	X	X	X	X
	Adherence to medications	MARS-10 questionnaire	X				X
Quality of life	Health-related quality of life	EQ-5D-5L questionnaire	X	X	X	X	X
Healthcare use and time off work (economic factors)	Primary and secondary care use over last 3 months	Bespoke question	X	X	X	X	X
	Time off work to seek care and/or due to asthma over last 3 months	Bespoke question	X	X	X	X	X
Psychosocial factors	Depression	PHQ-8 questionnaire	X				X
	Anxiety	GAD-7 questionnaire	X				X
	Self-efficacy	General Self-Efficacy Scale	X				X
	Smoking status	Bespoke question	X				X
OHC use factors (only related to intervention arm participants)	Amount and type (passive vs active) of OHC engagement	Bespoke question					X
Internet use for health support	eHealth literacy	eHealth Literacy Scale	X				
	Interest in and appropriateness of digital social interventions in primary care	Bespoke question	X				

* At the time of the study-related face-to-face appointment.

† Interim follow-up at 3, 6 and 9 months following the face-to-face appointment, data collected over the telephone by the research team.

‡ Final follow-up survey 12 months following the face-to-face appointment, survey self-completed by participants.

§ Primary outcome.

ACT, asthma control test; EQ-5D-5L, EuroQol 5-Dimensions 5-Level version; GAD-7, Generalised Anxiety Disorder 7-item scale; MARS-10, 10-item Medication Adherence Rating Scale; OHC, online health community; PHQ-8, 8-item Patient Health Questionnaire depression scale.

**Table 2:** Non-self-reported outcomes of interest

Outcome	Data source	Baseline*	12 months†
Number of asthma exacerbations over last 12 months	Discovery‡ and/or NHS Digital§	X	X
Primary and secondary care health service utilisation and associated costs over last 12 months	Discovery‡ and/or NHS Digital§	X	X
OHC engagement metrics (ie, amount of engagement, communities joined, number of logins, number of likes and time spent on pages), public posts and metadata, including time of post, thread and user details (outcome only related to intervention arm participants)	ALUK OHC data (provided by the manager of the OHC platform)		X

* At the time of the study-related face-to-face appointment.

† 12 months following the face-to-face appointment.

‡ Discovery is a clinical partnership project in East London to link primary and secondary care records, by creating a single database.

§ NHS Digital is a national provider of health-related data setting out to transform and improve healthcare in the UK.

ALUK, Asthma+Lung UK; NHS, National Health Service; OHC, online health community.

Patient data at baseline and interim follow-up will be collected and recorded directly in the online study database by the HCPs carrying out the face-to-face appointment and the research team, respectively. The final follow-up survey 12 months after the face-to-face appointment will be self-completed by participants (completion should take approximately 10 min).

### Exit interviews

At the end of the study, patients and HCPs involved will be invited to participate in a one-to-one, semi-structured, virtual exit interview. A convenience sampling approach, based on patients and HCPs who are available and willing to take part at the end of the study, will be used. Recruitment will continue until data saturation. Invitations will be made through a phone call/email/text message by the research team. A mutually convenient time for the interview will be agreed. If interviewees wish, reminder phone calls/emails will be made/sent.

Interviews will be undertaken by members of the research team and/or by appropriately trained members of the Centre for Applied Respiratory Research, Innovation and Impact (CARRii) patient and public involvement (PPI) group, remotely via Zoom or Microsoft Teams. Each interview will last up to 60 min. An interview schedule will be used (online supplemental material 2). In these interviews, we will explore patient and HCP views about their involvement in the study and receiving/delivering the intervention. All interviews will be digitally recorded, subject to consent, through recording functions in Zoom/Teams platforms.

**Figure 2:**
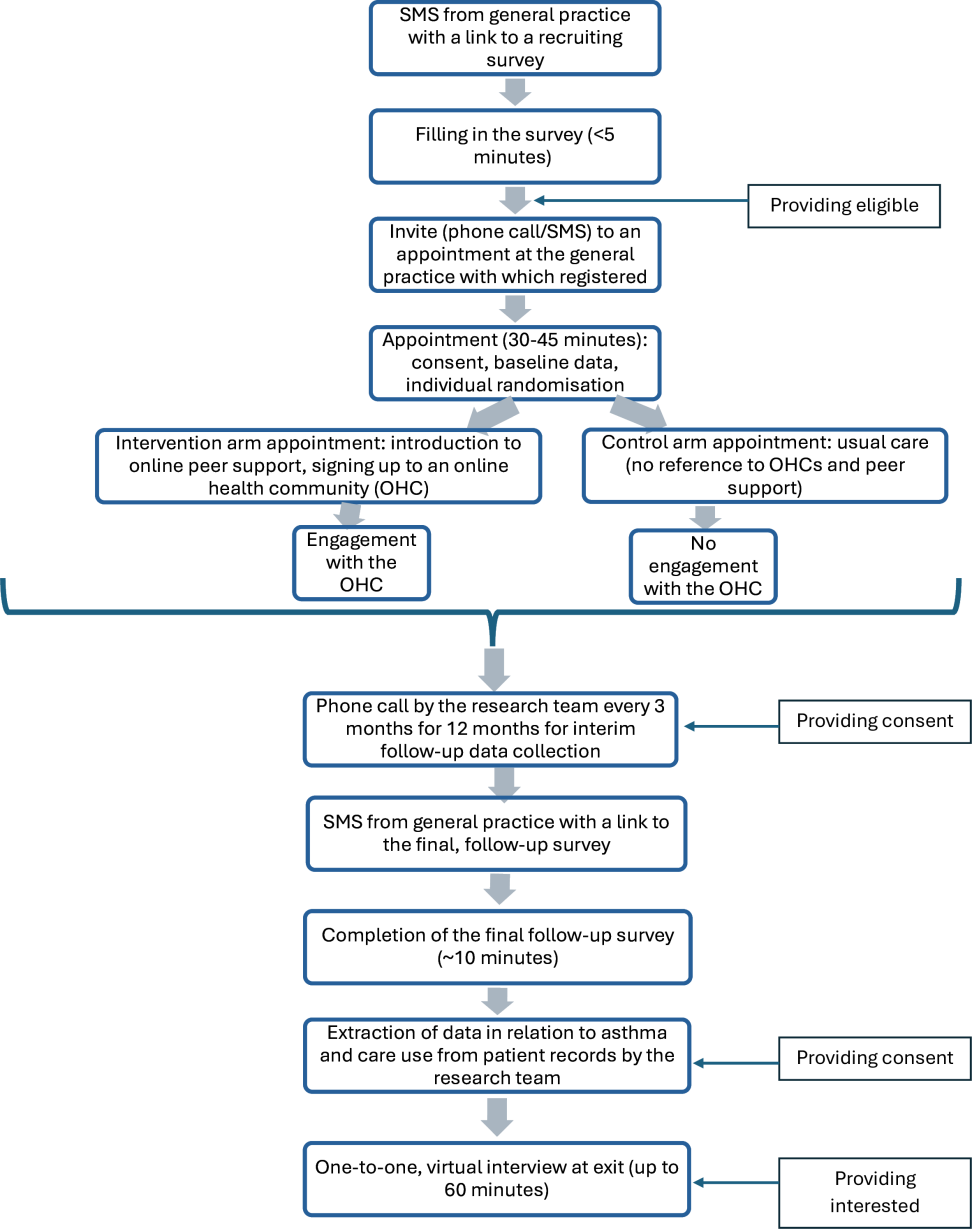
Flow diagram summarising the procedures each participating patient will undergo in this study.

Figure 2 provides an overview of the procedures each participating patient will have to undergo in this study.

### Sample size justification

It is estimated that a sample size of 586 patients (293 per arm, allowing 20% loss to follow-up) is needed for 90% power to detect a standardised effect size of 0.3 (equivalent to a difference from baseline to 12-month follow-up in ACT score of 1.05), assuming an SD of 3.5.^41^ This gives a total of 234 patients per arm with completed follow-up data. In the previous feasibility study, we had a 10% response rate in the recruiting survey and a recruitment rate (ie, proportion of survey respondents consenting to and receiving the intervention) of 9.8%. Assuming response and recruitment rates are similar in the RCT, the survey will need to be circulated to just over 59 000 patients (see figure 1 for detailed calculation).

### Data management

An online database will be designed on REDCap by the Pragmatic Clinical Trials Unit (PCTU) at Queen Mary University of London (QMUL), for study data capture and storage. Online copies of the recruiting survey, consent forms and case report forms for collecting baseline and follow-up measures will be stored in the online database. Data related to participants’ online activity in the ALUK OHC will be transferred by HealthUnlocked directly into the Data Safe Haven (DSH) platform (ie, QMUL’s technical solution for storing, transferring and analysing research information containing highly confidential data). The DSH will also be used to transfer the email addresses of patients having received the intervention to HealthUnlocked, so that their activity can subsequently be identified. Data extracted from clinical records will be stored on the DSH. Demographic data from HCPs participating in the exit one-to-one interviews will be pseudonymised and stored on the DSH. Any hard copies of demographic forms will be stored separately inside locked filing cabinets, within a locked office at QMUL. Original audio recordings from exit interviews will be downloaded and stored, along with original transcripts, on the DSH at QMUL. Edited versions of the transcripts, pseudonymised and with all identifiers removed, will be stored on QMUL’s secure server (SharePoint). Only members of the research team will have access to study data, which will be retained in accordance with QMUL policies. Processing and analysis of data will be undertaken solely on QMUL password-protected computers.

### Data analysis

All analyses of study data will be carried out collectively by the research team with extensive input from staff from the PCTU when it comes to statistical tests and health economic processes. Social network analysis of participants’ engagement with the OHC will be conducted using Python.^42^ Qualitative analyses will be facilitated by NVivo software.^43^ R^44^ and Stata^45^ will be used to carry out quantitative analyses.

### Quantitative analyses

Team members involved in developing the statistical analysis plan will be blinded to allocation of codes being provided to the analysis. The mean, median, range and SD for ACT at baseline and all follow-up time points will be presented along with the same for 8-item Patient Health Questionnaire depression scale, EuroQol 5-Dimensions 5-Level version (EQ-5D-5L), Generalised Anxiety Disorder 7-item scale, Medication Adherence Rating Scale, self-efficacy scores, number of asthma exacerbations and primary and secondary care health service utilisation over the last 12 months (at time points where these are collected).

The main analysis of the primary outcome will draw on a linear regression approach to compare the ACT at the final (12-month) follow-up point in the two groups, adjusting for baseline value, including general practice as a random effect in a mixed model. We will also include a repeat measures analysis (simultaneously analysing ACT score at each time point) to complement the primary analysis, again using a mixed-model procedure. In addition, we will carry out a per-protocol analysis, including in the treatment arm only those participants who used the digital intervention at least once in each of three consecutive months.

Similarly to the main analysis, we will compare 12-month data for the other outcomes listed above, adjusting for baseline using a mixed modelling approach. The exact nature of a given analysis will depend on the outcome variable (eg, continuous, binary, ordinal, categorical), allowing for GP-level clustering in the data.

Data related to healthcare use from healthcare records (eg, healthcare needs, including primary care visits, investigations, asthma medication, asthma exacerbations and hospital admissions) will also be analysed through descriptive and inferential statistics and assessed against baseline and follow-up measures. For example, regression models will also be employed to associate participant-reported outcomes and data in relation to primary/secondary care use, thereby further contributing to evidence regarding the effectiveness of the intervention.

### Health economic assessment

The health economic assessment will include within-trial and long-term cost-effectiveness analyses (likely a Markov model), which will be informed by the within-trial analysis, of OHCs’ enhanced management versus current standard of asthma management in UK primary care. The analysis will be conducted from the perspectives of both health and social care services and wider society, adhering to the National Institute for Health and Care Excellence guide to the methods of technology appraisal,^46^ to ensure that study findings are informative for national-level policy considerations.

As part of the health economic assessment, we will assess the resource requirements and costs for development, moderation, governance and clinical oversight of OHCs. Costs of OHCs, healthcare and other resource use will be evaluated using nationally representative unit costs of primary and secondary healthcare services.^47^ Participants’ quality of life and utility scores will be used in a regression model to examine the relationship between baseline characteristics, asthma control (ACT score) and adverse events during the study, with their impact on quality of life and utility. The within-trial cost-effectiveness will be assessed as incremental cost per quality-adjusted life year (QALY) gained, with QALYs assessed in two ways. First, study data will be used to directly evaluate individual participants’ QALYs over the duration of the study (primary analysis). Second, the quality-of-life regression model will be used to predict quality of life of participants during the study, to increase the precision in the analysis (secondary analysis).

The long-term cost-effectiveness of the OHCs will be evaluated using a long-term asthma model. The quality-of-life regression model will be used to project quality of life associated with different patients and model states in the model. This model, together with trial participants’ characteristics and effects of OHCs on the primary outcome, the ACT score and the adverse events during the study will be used to evaluate long-term cost-effectiveness of OHC in terms of incremental cost per QALY.

Efforts will be made to minimise omissions in prospectively collected data during the study (eg, ACT score, asthma exacerbations, EQ-5D-5L, some resource use). The impact of any missing data on health outcomes and costs will be addressed using appropriate methods depending on pattern and proportion of missing data and type of outcome measure. It is expected that linked routinely collected health data (eg, asthma exacerbations) may alleviate some missing data issues. Results will be presented as incremental cost-effectiveness ratios. The non-parametric bootstrap approach will be used to evaluate the uncertainty in cost-effectiveness while capturing the correlation between health outcomes and costs at individual level and cost-effectiveness acceptability curves will be used to summarise the uncertainty across thresholds of willingness to pay from £0 to 50 000 per QALY gained.

Both the statistical and health economic elements of the quantitative analysis will be described in more detail in a statistical analysis plan and health economic analysis plan, respectively, both of which will be completed and signed before the study database is formally locked.

### Analysis of qualitative data from exit interviews

Audio-recordings from the exit interviews will be transcribed *verbatim* by a professional transcribing agency and analysed thematically using the six stages of reflexive thematic analysis by Braun and Clarke.^48^ Both inductive and deductive approaches will be employed in thematic analysis coding. Coding schemes as well as themes and subthemes will be informed by social support theory (as framed by Dennis^49^) and will aim to synthesise and interpret experiences of the intervention’s delivery and participation in the study in general. Attention will be paid to identifying ‘direct’ effects (ie, directly impacting health-related outcomes), ‘buffering’ effects (ie, reducing the detrimental influence of stressors on health) and ‘mediating’ effects (ie, indirectly influencing health via cognitions, emotions and behaviours) in the interview data. These represent the main ‘effect models’ (ie, mechanisms resulting in beneficial outcomes) underpinning peer support interventions, as outlined in Dennis’s model.

### Analysis of OHC activity data

Data related to participant activity in the ALUK OHC will be analysed through qualitative (eg, thematic and content analysis) and quantitative techniques (eg, descriptive and inferential statistics) as well as network analysis methods (eg, network measures and visualisations) and sentiment analysis (eg, sentiment labelling and tracking sentiment dynamics). Analyses of this data will aim to generate meaningful themes (eg, themes related to self-management support and patterns of online communication leading to improved sentiment and self-management behaviours); quantify online peer support received versus that offered within the OHC environment; develop visual maps of the network containing key users including the participants and of information diffusion within that network and understand correlations between engagement with other peers and outcomes. For thematic analysis, the method of Braun and Clarke^48^ will be used and both inductive and deductive approaches to coding will be pursued. Should certain themes related to self-management behaviours be identified, the frequency of these concepts will be further explored/quantified in the whole data set via conceptual forms of content analysis. Theoretical mediators of the intervention effect will also be measured. OHC activity data will be linked to health-related data and Dennis’s conceptual model will be employed to link improvements in patient outcomes to theoretical mechanisms.^49^ For the network analysis, dynamic networks will be constructed and visualised to show the evolution of users’ activities in the OHC. Key network properties will be investigated using traditional centrality measures. In addition, ego-centred networks will be constructed, and Burt’s measures of structural holes and brokerage^50^ will be applied to these networks to provide in-depth understanding of users’ social capital. Sentiment analysis will explore how interactions, particularly with superusers, drive sentiment shifts, improved emotional well-being and behavioural changes, helping to uncover potential causal links between engagement patterns and users’ health-related outcomes. Finally, regression analyses will be conducted to unveil the correlation between network properties and users’ health-related outcomes.

### Timelines

The study is expected to take approximately 24 months to complete, including recruitment, face-to-face appointment delivery, follow-up data collection and exit interviews. The study is expected to commence in early spring 2025.

### Patient and public involvement

Τhe aim and research questions have been extensively informed by PPI work, including regular consultations with the PPI group of the CARRii (formerly known as Asthma UK Centre for Applied Research) and a public engagement activity funded by QMUL Centre for Public Engagement. PPIs reflected on the brevity of contacts with HCPs, highlighted the need for an intervention delivered by primary care HCPs that fosters norms and values in OHC engagement and described the potential of peer support through OHCs when no other sources of support are available. Some PPIs stated that they would not consider discussing their condition in an OHC, a factor considered when planning recruitment. The precise content of our intervention was designed collaboratively with primary care patients and clinicians.^39^ PPI input was also sought for the development of study documents (eg, recruiting survey, PIS, topic guide for exit interviews).

Two PPI representatives (BD and one other who is not a coauthor) will join the Independent Steering Committee (see ‘Study committees’ section). Members of the CARRii PPI group will provide regular feedback on drafted documents, approaches, governance issues and outcomes. PPI consultations will also take place during our regular CARRii monthly PPI meetings to discuss the progress of the study and interpret findings. PPIs may be offered training to assist with the conduct of exit interviews. A dissemination plan will be co-developed with the CARRii PPI group and PPI groups from charities and industry to highlight the project and its potential implications for the public. All PPIs will be reimbursed in accordance with NHS policies^51^ and NIHR guidelines.^52^

### Study committees

The sponsor (QMUL) retains the right to audit any part of the study. Study progress will be monitored by two committees. First, the Independent Steering Committee will be assembled in accordance with NIHR guidelines and will provide overall expert external supervision. It will meet annually, with additional ad hoc meetings held if necessary. The Committee consists of an independent chair, an independent clinician with relevant expertise, a statistician and a patient with asthma, all based in the UK. Second, the Project Management Group, comprising all coauthors and PCTU staff, will meet monthly to review progress against milestones and address any issues that arise.

### Ethics and dissemination

####  Ethical approval and issues

The study has been reviewed and given a favourable opinion by the NHS North East-Newcastle and North Tyneside 1 Research Ethics Committee (reference: 25/NE/0006) and the Health Research Authority (Integrated Research Application System Project ID: 349517).

Ethical and information governance matters associated with primary care HCPs promoting online peer support and actively signing patients up to OHCs are highlighted elsewhere.^36^ Risks for participants associated with this study are minimal. Participation will be voluntary, with no coercion or pressure on participants during recruitment. Participants will have the right to withdraw from the study at any point, without giving a reason and without consequence. All possible accommodations will be made to minimise the impact on participants’ work/other commitments. Participants will be reimbursed for their time (ie, £10 for attending the face-to-face appointment, £5 for completing the follow-up survey and £30 for participating in an exit interview).

Digital literacy/access is a potential issue as interest in OHCs and thus participation may entail self-selection bias and potentially exclude certain demographics (eg, older people, non-English-speakers or those whose first language is not English).

Receipt of the intervention will not affect the normal treatment or care that the patient would have otherwise received. Being in the intervention group will require patients to sign up to the ALUK OHC. The ALUK OHC is well-established and moderated by HCPs and some expert patient users. Our previous research found patients in the ALUK OHC being aware of limits of their expertise, allotting medical management tasks to HCPs.^53^

### Consent-related matters

All participants will have the opportunity to ask any questions they have at any stage, by directly contacting the research team. Completion of the recruiting survey will imply consent to take part in that survey and be contacted regarding participation in the RCT if eligible. Consent for participation in the RCT will be collected via an online form at the start of the face-to-face appointment with the HCP, once the HCP has established the patient’s capacity and before randomisation. This consent form (online supplemental material 3) will cover participation in the trial, collection of baseline and follow-up measures, analysis of data related to activity in the ALUK OHC, including disclosure of their email address to HealthUnlocked so that their OHC activity can be identified and provided to the research team, and access to primary care and hospital records. HCPs will also add a code on the online clinical records indicating participation in our study (provided the participant consents to this).

Consent for participation in the exit interviews (including permission to audio-record the interview) will be collected via an online consent form (online supplemental material 4) sent to participants along with the link to the virtual interview. Participants will have the option to complete the online consent form either at the time of the interview or beforehand.

### Adverse events

Due to the nature and design of this study, safety reporting of adverse events will not occur. No adverse events were recorded in the feasibility study. Adverse events occurring between questionnaires will not be recorded or reported as they are not the aim or focus of this study. Nevertheless, in the unlikely event that any adverse events come to our attention, these will be reviewed and reported in accordance with the sponsor’s requirements. The insurance that QMUL has in place provides cover for the design and management of the study as well as ‘No Fault Compensation’ for participants, which provides an indemnity to participants for negligent and non-negligent harm.

### Dissemination of research findings

Dissemination activities will take place throughout the programme of work, with results shared with key stakeholders. Findings will be disseminated via journal publications, presentations at academic and professional conferences, reports and briefings (eg, for policy makers), newsletters to participating practices and patients, webinars, traditional media and social media, workshops (eg, GP forums and informal 1:1 meetings), co-applicants’ professional networks and links with guideline groups. The CARRii PPI group (including our PPI coauthor BD) will advise on how best to reach and engage with the public and will disseminate findings through the group’s avenues. ALUK will support dissemination and promotion of our findings to their patient and scientific audiences using their digital and social media platforms (ie, Asthma Update email ~70 000 recipients; Facebook ~40 000 followers; Twitter ~30 000 followers). Our industry partner, HealthUnlocked, will contribute to the dissemination of findings through their media channels. We will also seek advice and help from the QMUL Press Office and the NIHR Communication Team.

Participants will be informed about the study results, but not at the individual level. Once research outputs are submitted for publication in peer-reviewed journals, executive (lay) summaries will also be prepared. These summaries will be disseminated to the participating general practices, which will be asked to disseminate findings through their own communication channels, as appropriate. No participants’ names will be disclosed during the dissemination process.

## Supplementary material

10.1136/bmjopen-2025-104367 online supplemental file 1

10.1136/bmjopen-2025-104367 online supplemental file 2

10.1136/bmjopen-2025-104367 online supplemental file 3

10.1136/bmjopen-2025-104367 online supplemental file 4

## Data Availability

Data are available on reasonable request.
