# Supplementary material for: Randomised controlled trial to measure effectiveness and cost-effectiveness of a digital social intervention promoted by primary care clinicians to adults with asthma to improve asthma control: protocol
Source: BMJ Open. 2025 Sep 12;15(9):e104367. doi: 10.1136/bmjopen-2025-104367 (PMC12434742; doi:10.1136/bmjopen-2025-104367)
Supplement: online supplemental file 1 [file bmjopen-15-9-s001.pdf]

AD HOC
Peer support for people with troublesome asthma

Queen Mary University of London

AD HOC Survey Case Report Form

[Please note that text in red is instructions/information for database development and should not be shown to the participant]

AD HOC: Screening

AD HOC: Participant Information Sheet

Please download and read through the full participant information sheet (PIS) for the AD HOC study below:

[Attachment: PDF of AD HOC Survey PIS]

Please note that clicking on this link might take you away from the survey homepage (depending on your browser). You can return to the survey homepage by clicking the back button on your browser.

If you are happy to proceed to complete the survey, please confirm below:

Please only proceed to complete the survey if the following statements are true for you:

1. I have a diagnosis of asthma. ☐ Yes ☐ No
2. I am 18 or older. ☐ Yes ☐ No

[must be YES to continue; if NO to any question, show text below]

You are not eligible to take part in the survey. The survey will now close.

[If YES to both questions;]

I would like to proceed to complete the survey ☐

[must be checked to continue]

You should have been invited via SMS text message sent from your GP surgery or via an advertising poster or video seen at your GP surgery. Please confirm this by selecting:

1. Your area/region [select from dropdown list; create holding places for up to 6]
2. Your GP surgery [select from dropdown list for surgeries within that region, i.e. list shown depends on answer above; create holding space for up to 15]

[cannot proceed if GP surgery not selected]

AD HOC: Survey (1)

Part 1: About your asthma symptoms

How would you describe your asthma?

- ☐ Mild
- ☐ Moderate
- ☐ Severe

[required, only one answer allowed]

[Asthma Control Test] This section is about your asthma symptoms. For each question, select the ONE answer from the list that best applies to you.

In the past *4 weeks,* how much of the time did your *asthma* keep you from getting as much done at work, school or home?

- ☐ All of the time
- ☐ Most of the time
- ☐ Some of the time
- ☐ A little of the time
- ☐ None of the time

[required, only one answer allowed]

During the past *4 weeks,* how often have you had shortness of breath?

- ☐ More than once a day
- ☐ Once a day
- ☐ 3 to 6 time a week
- ☐ Once or twice a week
- ☐ Not at all

[required, only one answer allowed]

During the past *4 weeks*, how often did your *asthma* symptoms (wheezing, coughing, shortness of breath, chest tightness or pain) wake you up at night or earlier than usual in the morning?

- ☐ 4 or more nights a week
- ☐ 2 or 3 nights a week
- ☐ Once a week
- ☐ Once or twice in the past 4 weeks
- ☐ Not at all

[required, only one answer allowed]

During the past *4 weeks,* how often did you have to use your rescue (blue) inhaler or nebuliser medication?

- ☐ 3 or more times a day
- ☐ 1 or 2 times a day
- ☐ 2 or 3 times a week
- ☐ Once a week or less
- ☐ Not at all

[required, only one answer allowed]

How would you rate your *asthma* control over the past *4 weeks?*

- ☐ Not controlled at all
- ☐ Poorly controlled
- ☐ Somewhat controlled
- ☐ Well controlled
- ☐ Completely controlled

[required, only one answer allowed]

[sum answers to the 5 ACT questions to calculate ACT score]

Part 2: About managing your asthma

This section is about support with managing your asthma. Who helps you (when needed) with your asthma in relation to the following tasks? For each task, please tick as many boxes as apply to you.

Who helps you with taking your medication and attending medical appointments for asthma?

- ☐ General practitioner
- ☐ Practice nurse
- ☐ Pharmacist (in GP surgery and/or in high street pharmacy)
- ☐ Other clinician
- ☐ Family
- ☐ Best friend
- ☐ Friends
- ☐ Asthma support group
- ☐ Somebody else
- ☐ No-one/Don't need any help

[required]

Who helps you with adapting your lifestyle and life roles to your asthma?

- ☐ General practitioner
- ☐ Practice nurse
- ☐ Pharmacist (in GP surgery and/or in high street pharmacy)
- ☐ Other clinician
- ☐ Family
- ☐ Best friend
- ☐ Friends
- ☐ Asthma support group
- ☐ Somebody else
- ☐ No-one/Don't need any help

[required]

Who helps you with processing emotions that arise from having asthma, for example, when feeling unwell?

- ☐ General practitioner
- ☐ Practice nurse
- ☐ Pharmacist (in GP surgery and/or in high street pharmacy)
- ☐ Other clinician
- ☐ Family
- ☐ Best friend
- ☐ Friends
- ☐ Asthma support group
- ☐ Somebody else
- ☐ No-one/Don't need any help

[required]

Who are your most frequent sources of help with your asthma?

- ☐ General practitioner
- ☐ Practice nurse
- ☐ Pharmacist (in GP surgery and/or in high street pharmacy)
- ☐ Other clinician
- ☐ Family
- ☐ Best friend
- ☐ Friends
- ☐ Asthma support group
- ☐ Somebody else
- ☐ No-one/Don't need any help

[required]

AD HOC: Eligibility

Part 1: Interest in taking part in research

Would you be interested in taking part in our research study, in which you might be offered an intervention designed to help people with troublesome asthma? The intervention involves being signed up with an online health forum for people with asthma (selecting YES is not a commitment to take part).

☐ Yes ☐ No

[required, if YES go to next question; if NO, go to exit page]

Are you already a member of the Asthma + Lung UK online health forum or other online asthma forums, including Facebook groups for asthma patients?

☐ Yes ☐ No

[required, if NO go to next question; if YES, show text below then go to exit page]

As you are already a member of an online forum for people with asthma you are not eligible to take part in our research study.

Thank you for your interest in our research.

Eligibility

[If ACT is <20]

You seem to be having some trouble with asthma symptoms and as you're not already a member of an online forum for people with asthma, you are eligible to take part in our research study.

Please provide your name, email address AND mobile number, and postcode so we can contact you about taking part. You will be contacted by a member of the research team or a nurse from your GP surgery. This is not a commitment to take part – we will give you more information about the study then you can decide whether to take part or not. Your contact details will not be shared with anyone else and will be securely handled, according to Data Protection regulations.

First name: ……………………

[required]

Last name: ………………………

[required]

Email address: ……………………………………………………………

[required, must be an email address]

Mobile phone number: …………………..

[required, must have 11 digits, starting 07]

Postcode: …………………..

[required]

[If ACT is ≥20]

Your asthma appears to be well controlled. Because the intervention is for people with troublesome asthma, you are not eligible to take part in our research study.

Thank you for your interest in our research.

[go to exit page]

AD HOC: Exit page

Thank you for completing this survey. We very much appreciate you taking the time to contribute to our research study.

If you have provided your contact details, we will be in touch soon about the next stage of the study.

If you have any questions, or concerns, about this research study, please contact us at:

AD HOC Research Team
Queen Mary University of London
Yvonne Carter Building
58 Turner Street
London
E1 2AB
Email: adhoc@qmul.ac.uk
Tel: 07916 136187

You can also find further information about our research on the AD HOC Study website www.qmul.ac.uk\ADHOC

If you do not wish to participate further but want help for your symptoms, please speak to your GP. More support and information can also be found on the Asthma+Lung UK website: www.asthmaandlung.org.uk

FUNDED BY
NIHR | National Institute for Health Research

Queen Mary University of London
