# Supplementary material for: Randomised controlled trial to measure effectiveness and cost-effectiveness of a digital social intervention promoted by primary care clinicians to adults with asthma to improve asthma control: protocol
Source: BMJ Open. 2025 Sep 12;15(9):e104367. doi: 10.1136/bmjopen-2025-104367 (PMC12434742; doi:10.1136/bmjopen-2025-104367)
Supplement: online supplemental file 2 [file bmjopen-15-9-s002.pdf]

Peer support for people with troublesome asthma

Exit Interview Schedule (Patients and healthcare professionals)

Introduction

- Interviewer to introduce themselves.
- Explain the aim of the interview.
- Explain the process (highlight confidentiality).
- Answer any questions the participant has.
- Ask the participant to sign the Consent Form (if not already done online).
- Start the recording (after confirming consent) and start the interview.
- Ask the participant to introduce themselves and repeat confirmation of consent for recording.

Questions

- **Experience of delivering/attending the study-related consultation**
    - **HCPs**: What was your experience of the online training? Any additional training needs that weren't covered?
    - **HCPs**: Was there additional benefit from meeting with a researcher in person, e.g. to discuss the study procedures? Was this useful?
    - **HCPs**: What was your experience of contacting patients and arranging the consultation? Time consuming? What went well (or didn't)?
    - **HCPs**: What was your experience of delivering the consultation? What did you like/not like? Time requirements? Consent procedure – any issues?
    - **HCPs**: How did you find the data collection/entry process? Did you complete it online during the consultation or on paper? Had you used REDCap or any other online study database before?
    - **HCPs**: How did you find the randomisation tool/process?
    - **HCPs**: What was your experience of patients not wanting to take part or withdrawing? (were there any, what were their reasons?)

- **All patients**: What was your experience of attending the consultation? Did you receive the intervention (get signed up to the online asthma forum) or not? What did you like/not like? Time requirements? Consent procedure – any issues?
- **Control patients**: Were you happy to take part knowing you are not receiving the intervention? Why?
- **Intervention patients**: How was the process of signing up with the asthma forum? Information absorbable or complicated? Any suggestions to improve and simplify?
- **All patients**: Did you complete the questionnaires (in the consultation) online or on paper forms? How was that? Any suggestions for improvements?

- **Experience of the Asthma + Lung UK asthma forum**
  - **HCPs**: Did you have a chance to look at the forum, browse around it? What did you think? Would it be useful for patients? How did you find the process of demonstrating the forum to the patients during the consultation?
  - **Intervention patients**: How did the nurse/person delivering the consultation encourage you to engage with the forum?
  - **Intervention patients**: Have you engaged with the forum during the follow-up period (last 12 months) and, if so, how have you used it (just reading, replying to posts, starting new threads, private messages)? How much/how often have you used it?
  - **Intervention patients**: Was it helpful to receive monthly text messages from the study team during the follow-up period to remind you about using the forum?
  - **Intervention patients**: Have you found the information in the forum useful? Any issues with any information/advice from the forum?
  - **Intervention patients**: Do you think you will continue to use the forum after the end of this study?
  - **Intervention patients**: What barriers are there to engaging with the forum and how could these be overcome?
  - **Intervention patients**: How does participation in a study like this impact engagement with an online forum (e.g., does it promote/hinder engagement)?

- **Intervention patients**: Do you think this intervention would be useful to a lot of asthma patients? Would you recommend it to others? How could it be made more widely available (e.g., via GP surgery, asthma review, any other suggestions)
- **Control patients**: Did you sign up to the asthma forum yourself? If so, when? Was it after the follow-up? (if they did sign up, ask questions above, as relevant)

- **(All patients) Experience of follow-up data collection**
  - How was the process of completing the follow-up questionnaire? Any issues?
  - How would you prefer to access the questionnaire? (link via email or text message; ok to receive text message reminders to complete?)
  - Did you receive calls every three months from the research team during the follow-up period? If yes, how was that? Were you happy to receive the calls? Any issues with the questions?
  - Were you happy for your activity/use of the asthma forum to be analysed by the researchers? Did you think about it? Did it put you off from using the forum?

- **Asthma symptoms and control during the study**
  - How was your asthma during the study follow-up (last 12 months)? Any changes/improvements?
  - **Intervention patients**: Do you think engaging with the asthma forum made any difference to your asthma symptoms/how you manage your asthma? If so, how?

Ending the interview

- Ask the participant if they have anything else to add (then stop recording).
- Inform them how they can reach the research team if they have more questions.
- Thank them – let them know how helpful they have been.
- Ask HCPs for the address to send the gift card to (if not already provided) as compensation for their time (note that address is only for this purpose – information will not be retained).
