# Supplementary material for: Randomised controlled trial to measure effectiveness and cost-effectiveness of a digital social intervention promoted by primary care clinicians to adults with asthma to improve asthma control: protocol
Source: BMJ Open. 2025 Sep 12;15(9):e104367. doi: 10.1136/bmjopen-2025-104367 (PMC12434742; doi:10.1136/bmjopen-2025-104367)
Supplement: online supplemental file 3 [file bmjopen-15-9-s003.pdf]

Peer support for people
with troublesome asthma

AD HOC Trial: Consent Form

[Please note that text in red is instructions/information for database development and should not be shown to the participant]

Consent Form (part 1/3)

Study Participant ID ***[created from survey, feeds through, 4 digits]*** ☐

Please note agreement with the following statements is compulsory to complete your consent to the study

1. I confirm that I have read and understood the information sheet [**version 2.0, dated 17.02.2025**] for the above study; or it has been read to me. I have had the opportunity to consider the information, ask questions and have had these answered satisfactorily.<br><br>*If you have not reviewed the information sheet for the AD HOC Trial it can be downloaded using the link below. Please note that clicking on this link might take you away from the survey homepage (depending on your browser). You can return to the survey homepage by clicking the back button on your browser.*<br><br>***[Attachment: PDF of AD HOC Trial PIS]***<br><br>*[must answer YES]*	☐ Yes ☐ No
2. I understand that my participation is voluntary (my choice) and that I am free to stop taking part in the trial at any time without giving any reason, without my medical care or legal rights being affected. I understand that if my withdrawal occurs during or after data analysis, information I have provided up to that point will still be used in the research.<br><br>*[must answer YES]*	☐ Yes ☐ No
3. I understand that my data will be accessed by the research team to compare my health-related measures before and after the consultation (at the end of the follow-up period), thereby enabling	☐ Yes ☐ No

building of evidence in relation to the effectiveness of online peer support.<br>*[must answer YES]*	
4. I understand that my data will be securely stored at Queen Mary University of London, in accordance with the University's data protection guidelines, for 25 years.<br>*[must answer YES]*	☐ Yes ☐ No
5. I agree to share, with the research team and their partner managing the Asthma+Lung UK online health forum platform (HealthUnlocked), the email address I use to sign up (if randomised to the intervention group).<br>*[must answer YES]*	☐ Yes ☐ No
6. I understand that the researchers will not identify me in any publications and other outputs using personal information obtained from this trial.<br>*[must answer YES]*	☐ Yes ☐ No
7. I understand that the information collected about me may be used to support other research in the future, and it may be shared with other researchers, without my name/identifiable information being included.<br>*[must answer YES]*	☐ Yes ☐ No

Consent Form (part 2/3)

Please note agreement with the following statements is optional

8. I agree to the research team sending me monthly SMS text message reminders (which do not require a response).<br>*[must answer YES or NO]*	☐ Yes ☐ No
9. I agree to the research team phoning me every 3 months during the trial to collect information about my asthma symptoms and exacerbations.<br>*[must answer YES or NO]*	☐ Yes ☐ No
10. I agree that information about me held by my GP surgery and NHS Digital may be used to provide information about my health and treatments to the research team. To do this, my **NHS number,** along	☐ Yes ☐ No

with my **sex, date of birth, and postcode** will be shared with the relevant NHS organisation, allowing them to provide Queen Mary University of London with my de-identified personal data, which can only identify me by the study ID. Data will be stored in a secure data environment and only accessed by the ADHOC study team. I understand that the temporary computer files created for this purpose will be destroyed once the de-identified data has been transferred to Queen Mary University of London. *[must answer YES or NO]*	
11. I agree to my participation in the trial being recorded on my GP records so that the care team are aware of it. *[must answer YES or NO]*	☐ Yes ☐ No
12. I understand and agree that, if randomised to the intervention group, my activity in the online health forum (apart from private, one-to-one messages sent to other members) will be shared by HealthUnlocked with the research team for research-related purposes. [must answer YES or NO]	☐ Yes ☐ No

Consent Form (part 3/3)

Please note the following fields are compulsory to complete your consent to the study

13. I agree to take part in the AD HOC Trial. *[must answer YES]*	☐ Yes ☐ No

[all fields are required]

Participant name: [free text]

Participant electronic signature: [add signature box]

Participant signature date: [dd-mm-yyyy]

Name of healthcare professional taking consent: [free text]

Healthcare professional electronic signature: [add signature box]

Healthcare professional signature date: [dd-mm-yyyy]
