# Supplementary material for: Randomised controlled trial to measure effectiveness and cost-effectiveness of a digital social intervention promoted by primary care clinicians to adults with asthma to improve asthma control: protocol
Source: BMJ Open. 2025 Sep 12;15(9):e104367. doi: 10.1136/bmjopen-2025-104367 (PMC12434742; doi:10.1136/bmjopen-2025-104367)
Supplement: online supplemental file 4 [file bmjopen-15-9-s004.pdf]

AD HOC
Peer support for people with troublesome asthma

Queen Mary University of London

Consent Form (Exit Interviews)

Measuring whether promotion of a digital social intervention by primary care healthcare professionals and subsequent engagement with online peer support improves health and well-being of patients with asthma and is cost-effective: The AD HOC Trial

Study Participant ID

Thank you for your interest in this research.

Should you wish to participate in the study, please consider the following statements. Before signing the consent form, you should initial all or any of the statements that you agree with. Your signature confirms that you are willing to participate in this research, however you are reminded that you are free to withdraw your participation at any time.

Statement	Please initial box
1. I confirm that I have read and understood the information sheet [**version 1.0, dated 30.09.2024**] for the above study; or it has been read to me. I have had the opportunity to consider the information, ask questions and have had these answered satisfactorily.	
2. I understand that my participation is voluntary (my choice) and that I am free to stop taking part at any time without giving any reason, without my medical care or legal rights being affected. I understand that if my withdrawal occurs during or after data analysis, information I have provided up to that point will still be used in the research.	
3. I understand that data I provide during the interview will be accessed by the research team to enable analysis and extraction of meaningful conclusions.	
4. I understand that interview transcripts will be securely stored at Queen Mary University of London, in accordance with the University's	

data protection guidelines, for 25 years, in pseudonymised form. I also understand that audio-recordings will also be stored for the same duration and in accordance with data protection guidelines.	
5. I agree to the interview being audio-recorded [optional].	
6. I agree that quotes transcribed from my audio material may be published as part of this research using a coding system. I also understand that my identity will not be revealed in any publication or dissemination of findings.	
7. I understand that the researchers will not identify me in any publications and other outputs using personal information obtained from this study.	
8. I understand that the information collected about me may be used to support other research in the future, and it may be shared with other researchers, without my name/identifiable information being included.	
9. I agree to take part in the above study.	

_____________________ _____________ ______________________________

Participant name Date Signature

_____________________ _____________ ______________________________

Name of person taking consent Date Signature
