# Supplementary material for: Randomised controlled trial to measure effectiveness and cost-effectiveness of a digital social intervention promoted by primary care clinicians to adults with asthma to improve asthma control: protocol
Source: BMJ Open. 2025 Sep 12;15(9):e104367. doi: 10.1136/bmjopen-2025-104367 (PMC12434742; doi:10.1136/bmjopen-2025-104367)
Supplement: Supplementary file 5 [file bmjopen-2025-104367.reviewer_comments.pdf]

PEER REVIEW HISTORY

BMJ Open publishes all reviews undertaken for accepted manuscripts. Reviewers are asked to complete a checklist review form and are provided with free text boxes to elaborate on their assessment. These free text comments are reproduced below.

ARTICLE DETAILS

Title (Provisional)

A randomised controlled trial to measure effectiveness and cost-effectiveness of a digital social intervention promoted by primary care clinicians to adults with asthma to improve asthma control: protocol

Authors

Karampatakis, Georgios Dimitrios; Wood, Helen; Griffiths, Chris; Taylor, Stephanie; Toffolutti, Veronica; Bird, Victoria Jane; Lea, Nathan C; Ashcroft, Richard; Coulson, Neil; Panzarasa, Pietro; Li, Xiancheng; Sheikh, Aziz; Relton, Clare; Sastry, Nishanth; Watson, Jane S; Mant, Jonathan; Marsh, Viv; Day, Bill; Mihaylova, Borislava; Walker, Neil; De Simoni, Anna

---

VERSION 1 - REVIEW

**Reviewer**	**1**
**Name**	**Costello, Richard**
**Affiliation**	**Royal College of Surgeons Ireland, Respiratory Medicine**
**Date**	**07-Jul-2025**
**COI**	**None**

This is an important and already peer-reviewed and grant-funded study; therefore, my role is to address issues related to the study design and presentation of the approach. I am happy that this study protocol is clear and well described.

I have two comments, the first relates to the use of ACT to assess asthma control. While widely used, this tool is at least in my hands very highly influenced by co-existing non asthma-related conditions, such as anxiety, reflux and obesity. What this means, is that the score can remain low despite good asthma control (measured objectively with tools such as blood eosinophils and lung function). It is also highly correlated with EQ5D. On a minor point the score was bought by a company and the authors may have to pay to use the tool.

This moves to my second point. The ACT is a measure of self reported perception, it often does not match with healthcare use Would you consider objective measures of adherence, such as pharmacy and emergency pick up rates? Objective measures of health use will inform the cost-effectiveness of the intervention, which is very important. It will justify a national rollout, Would the team consider using this as the 1 endpoint

---

VERSION 1 - AUTHOR RESPONSE

Reviewer 1 comments	Authors' response
I have two comments, the first relates to the use of ACT to assess asthma control. While widely used, this tool is at least in my hands very highly influenced by co-existing non asthma-related conditions, such as anxiety, reflux and obesity. What this means, is that the score can remain low despite good asthma control (measured objectively with tools such as blood eosinophils and lung function). It is also highly correlated with EQ5D. On a minor point the score was bought by a company and the authors may have to pay to use the tool.	Thank you. We have added a new bullet point in the 'Strengths and Limitations' section to acknowledge the limitations of using ACT score as our primary outcome. This sentence reads (page 3, lines 102-105): '**The primary outcome of interest, Asthma Control Test (ACT), may be influenced by co-existing non-asthma-related conditions and can remain low despite good asthma control; therefore, we will triangulate ACT scores with other outcomes, especially asthma-related healthcare utilisation.**'<br><br>To the best of our knowledge, as this is solely university-led research, there are no fees for using the ACT tool. Nevertheless, we have submitted a query to 'Quality Metric' and will proceed accordingly once we receive their response (currently pending).

This moves to my second point. The ACT is a measure of self reported perception, it often does not match with healthcare use Would you consider objective measures of adherence, such as pharmacy and emergency pick up rates? Objective measures of health use will inform the cost-effectiveness of the intervention, which is very important. It will justify a national rollout, Would the team consider using this as the 1 endpoint.	Thank you. As described in the protocol, we aim to triangulate self-reported asthma-related healthcare utilisation with corresponding data extracted from healthcare records for participants who provide consent (see page 10, lines 303-322 and Tables 1 and 2 on pages 27-29).<br>We do not plan to use any pharmacy-related data (e.g., prescription data) due to time and resource constraints.
